# Formation and Development of Taproots in Deciduous Tree Species

**DOI:** 10.3389/fpls.2021.772567

**Published:** 2021-12-02

**Authors:** Paulina Kościelniak, Paulina Glazińska, Jacek Kȩsy, Marcin Zadworny

**Affiliations:** ^1^Institute of Dendrology, Polish Academy of Sciences, Kórnik, Poland; ^2^Department of Plant Physiology and Biotechnology, Faculty of Biological and Veterinary Sciences, Nicolaus Copernicus University, Toruń, Poland; ^3^Centre for Modern Interdisciplinary Technologies, Nicolaus Copernicus University, Toruń, Poland

**Keywords:** root architecture, trees, genes, transcription factors, hormones, miRNA, drought

## Abstract

Trees are generally long-lived and are therefore exposed to numerous episodes of external stimuli and adverse environmental conditions. In certain trees e.g., oaks, taproots evolved to increase the tree’s ability to acquire water from deeper soil layers. Despite the significant role of taproots, little is known about the growth regulation through internal factors (genes, phytohormones, and micro-RNAs), regulating taproot formation and growth, or the effect of external factors, e.g., drought. The interaction of internal and external stimuli, involving complex signaling pathways, regulates taproot growth during tip formation and the regulation of cell division in the root apical meristem (RAM). Assuming that the RAM is the primary regulatory center responsible for taproot growth, factors affecting the RAM function provide fundamental information on the mechanisms affecting taproot development.

## Introduction

Roots, functionally and structurally diverse, form an integrated system allowing for water and nutrient acquisition (Freschet et al., 2021a). Many aspects of root foraging are determined by differences in root types. The exploitation of soil water is primarily carried out by the smallest and most ephemeral roots, i.e., absorptive roots (McCormack et al., 2015, 2017). The development of taproots, allows for the production of absorptive roots in deep soil layers (Bleby et al., 2010; Mackay et al., 2020). Given the essential role of a plant’s root system, understanding the relationship between the root structure and function, should include an assessment of the relationship between taproot development and absorptive root formation. Together they play an important role in regulating water potential in plants and may also have significant consequences for the hormonal interactions and signaling described in the review hereafter.

Despite the important functions of taproots in many woody plants, significant questions remain on how internal and external factors control the growth and development of taproots. Genes, hormones, and microRNAs regulate every stage of root development (Petricka et al., 2012). However, it is unclear, if these regulating components interact with each other to control individual cell division, growth, and differentiation, and taproot development as a whole. Taproot development is determined at the embryonic stage, through the directed regulation of cell division and expansion, which is also influenced by external changes, e.g., soil moisture. Knowledge about signaling of internal and external factors is fundamental in understanding mechanisms responsible for taproot growth (Lynch et al., 2012). While the identification of key regulators of root growth is essential, it is also crucial to understand how these regulators interact. Major factors often achieve their function through an integrated effect on other, categorized as “composite factors” (Mitsis et al., 2020). On one hand, composite factors comprise different genes responsible for different individual, lower-level components (like the transcriptional, post-transcriptional, translational, and post-translational components), while on the other hand integrated growth involves “underlying factors” that vary in a coordinated manner as determined by pleiotropic or highly linked genes and/or tight hormonal control (Mitsis et al., 2020).

Our present objective is to determine individual and composite factors that affect and regulate taproot development and growth, and the influence of environmental stimuli on these factors. Such knowledge could contribute to the development of seedlings cultivation strategies, which further enabling taproot restoration in container-grown trees, e.g., oaks, in which taproots are typically rendered non-functional by air pruning. For example, long-term taproot pruning reduces the access of planted oaks to water during drought periods (Zadworny et al., 2014, 2019, 2021). First, we review, general information on taproot morphogenesis and function, especially with respect to tree response to drought, second we revisit changes in hormone-regulated root development, third we investigate genetic factors influencing root formation in deciduous plants.

## Characterization of the Taproot System

Commonly, the classification of roots is based on the position of emergence, and the recognition that most of the functional traits of root systems as a whole are directly related to this location (Zobel and Waisel, 2010; Zobel, 2011; Freschet et al., 2021b). Primary roots in tree seedlings, known also as a taproots, develop from the central embryonic root – the radicle, forming the central axis of a root system (Zobel and Waisel, 2010; Wang et al., 2014; Freschet et al., 2021b). The initial formation of taproots are important, allowing the root system to reach rapidly water at deeper soil depths, a factor that can be extremely important for trees exposed to periods of drought (Barbeta and Peñuelas, 2017; Zadworny et al., 2019; Mackay et al., 2020). However, taproots undergo dynamic process governing root system development and architecture, including the formation of lateral and absorptive roots (Clowes, 2000). The requirements for water and nutrients in plants change over time and therefore root systems must dynamically adapt to those changing needs when the rest of the plant grows bigger (Di Iorio et al., 2005). Thus, it is important to determine where, and how, changes of the environment are sensed and transduced into root development.

### Taproot Morphogenesis

**FIGURE 1:**
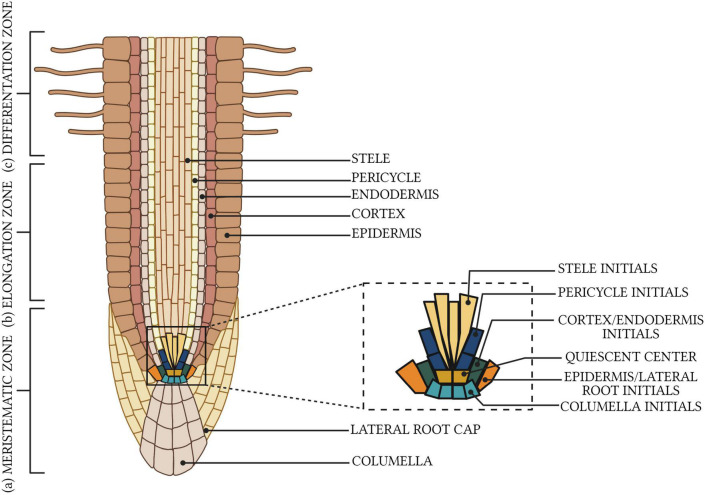
Organization of plant root meristem. There are three distinct developmental zones: **(a)** meristematic with visible the quiescent center and initial cells in a stem cell niche, **(b)** elongation, and **(c)** differentiation. Created with BioRender.com.

A comprehensive understanding of the root growth potential arises from the apical configuration of a primary root – a synonym of taproot root (Baluška et al., 2010; Freschet et al., 2021b). The ability of taproots to penetrate compact soil layers is due to the larger size of the root apex and the rapid elongation behind the root cap (Clowes, 2000), as taproot meristematic cells have a physiological and mechanical advantage over meristematic cells, compared to other root types, e.g., lateral roots (Perilli et al., 2012). Indeed, the pattern of postembryonic root development can be projected through an analysis of the initial cells located in the root apical meristem (RAM) cells (Figure 1; Perilli et al., 2012; Sozzani and Iyer-Pascuzzi, 2014). Ablation of the RAM in water-limited conditions results the formation of a highly branched, shallow root system (Dubrovsky and Gómez-Lomelí, 2003; Shishkova et al., 2013; Drisch and Stahl, 2015), indicating the essential role of a taproot in root system architecture (Dolan et al., 1993; Chapman et al., 2002; Sabatini et al., 2003) for water acquisition from deeper soil layers (Robbins and Dinneny, 2018; Gupta et al., 2020).

There are three unanswered questions remaining in the regard to the taproot root meristems: (1) how does organization and cellular signaling enable a taproot to grow and penetrate deep soil layers, (2) what internal factors enable taproots to grow rapidly and penetrate deep soil layers, and (3) how does soil water limitation induce the vertical growth of taproots. Aside from the unanswered questions above, how much does the genetic control the cell division explain the continued maintenance of root growth and apical dominance of taproot meristems (Perilli et al., 2012; Sozzani and Iyer-Pascuzzi, 2014). Current findings indicate that differences in inter-tissue signaling and the relationship between tissue-types are mostly responsible for matching meristem growth and root topology patterning (Peters and Tomos, 1996). Meristem enlargement, through increased cell division, and the transition of the cells into the expansion zone, occurs not only in response to internal stimuli during plant ontogenic development, but also in direct response to water supply (Benková and Hejatko, 2009; Mira et al., 2017). It seems likely, however, that cell division predominates cell differentiation in taproot meristems over the long-term to prevent the cessation of root growth until roots reach deep soil layers (Shishkova et al., 2008).

### Taproot Function in Deciduous Trees

Insufficient water availability and associated reduced water uptake by absorptive roots are the main factors contributing to global forest decline (Allen et al., 2015; Choat et al., 2018; Zadworny et al., 2021). Countering drought stress can be achieved by enhancing water acquisition and/or reducing water consumption, while increased root proliferation and taproot elongation increases water uptake from deeper soil layers (Arend et al., 2011; Tuberosa, 2012; Brunner et al., 2015). Mackay et al. (2020) reported that water acquisition in shallow soil layers declines as drought severity increases. Therefore, long taproots can improve water uptake, and help to compensate for increased water usage (Mucha et al., 2018; Skiadaresis et al., 2019), e.g., in oaks that produce a dominant taproot (Osonubi and Davies, 1981; Löf and Welander, 2004; Bréda et al., 2006; Mucha et al., 2018). Deep-rooted plants access water from deep soil layers and transport it to shallow, drier roots, increasing a plants’ ability to survive due to hydraulic redistribution process (Domec et al., 2004; Smart et al., 2005; David et al., 2013). Nevertheless, such watering is rather uncommon as shallow, fine roots are abandoned and die during the dry season in some drought-adapted tree species and grow back when water is available (Montagnoli et al., 2019). This raises the question, whether water limitation contrarily accelerates taproots growth into deeper soils in response to drought. Hormonal induced accumulation of osmoprotectant metabolites enabling root elongation during drought, confirms that this may be the case (Fàbregas et al., 2018). Therefore, a rigorous quantification of the components and molecular mechanisms regulating taproot growth in trees is required, especially among deciduous angiosperms, such as oak and chestnut, as deep taproots may determine resilience to drought. The first step in developing a mechanistic understanding of taproot growth would be to determine the regulatory effect of different phytohormones on cell division in the taproots RAM.

## Effect of Phytohormones on Root Growth

**FIGURE 2:**
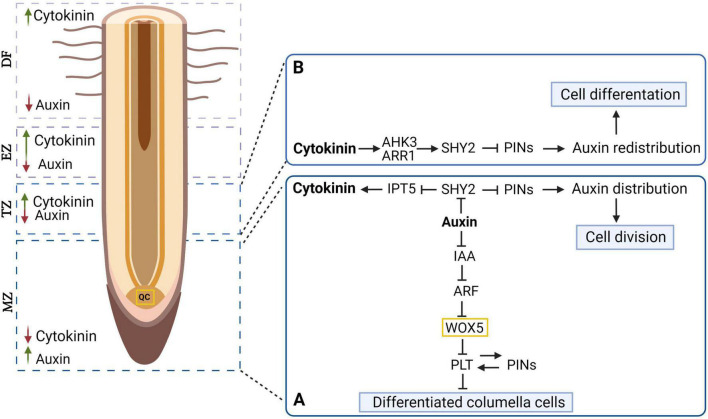
Mechanism of auxin and cytokinin interaction in root meristem development. The distribution of auxin and cytokinin in different lots of the longitudinal section of the tree root is shown (green arrow – higher level, red arrow – lower level). **(A)** Auxin mediating WOX5 (expressed in the quiescent center cells (QC) and PLT (expressed in the stem cells surrounding the QC) expression plays a key role in the differentiation of columella cells. Also, in the apical root meristem, auxin by degrading SHY2 proteins promotes the expression of PINs, which control the auxin gradient and subsequently affect cell division. **(B)** In contrast to the auxin, cytokinin inhibits PIN expression by promoting SHY2 expression, leading to auxin redistribution and cell differentiation. PLT, PLETHORA; PINs, PINFORMED; WOX, WUSCHEL RELATED HOMEOBOX 5; ARF, AUXIN RESPONSE FACTOR; IAA, INDOLE-3-ACETIC ACID; SHY2, SHORT HYPOCOTYL 2; IPT5, ISOPENTENYL-TRANSFERASE 5; AHK3, ARABIDOPSIS HIS KINASE 3; ARR1, ARR TRANSCRIPTION FACTORS; MZ, meristematic zone; TZ, transition zone; EZ, elongation zone; DF, differentiation zone, QC, quiescent centre [based on Su et al. (2011)]. Created with BioRender.com.

Root growth is also regulated *via* signal transduction pathways, including complex, environmental-sensing networks. The signaling pathways regulate plant root elongation, radial growth, branching, and overall architecture (e.g., root growth and development), and concomitantly water and nutrient uptake (Jung and McCouch, 2013; Ristova et al., 2018). Importantly, individual phytohormones do not regulate root growth and development independently, but rather function in an interactive manner (Figure 2; Xuan et al., 2016). Increased knowledge about these interactions may help to clarify the underlying mechanism regulating the pattern of taproot growth (Zhang et al., 2017). Hormones incidence and composition can contribute to improvements of taproot growth, and could contribute to its regeneration, when damaged as a consequence of root pruning during nursery cultivation. Thus, it is important to improve our understanding of specific hormones and their influence on the development and growth of taproots.

### Auxin

Auxin contributes to the positioning and formation of meristematic cells during organogenesis (Jiang and Feldman, 2010), as well as the retainment of mitotic activity in meristems (Beemster and Baskin, 2000; Galinha et al., 2007; Stepanova et al., 2008), as well as the fast elongation and differentiation of cells (Rahman et al., 2007; Benková and Hejatko, 2009; Ishida et al., 2010). Auxin accumulation in developing RAM cells has revealed that proteins, belonging to the PINFORMED family (PINs; PIN1, 2, 3, 4, 7), are necessary for the formation of an auxin gradient, and regulating the auxin distribution and acropetal transport to the root apex (Blilou et al., 2005). Auxin gradients that induce the expansion of cells and inhibit cell division in the extension zone (Blilou et al., 2005) by the expression of PLETHORA transcription factors (TFs) (Aida et al., 2004), may also regulate taproot elongation. The maintenance of root tip size and growth rate in transgenic Arabidopsis mutants in which PIN genes were silenced, provided evidence that the formation of an internal auxin gradient is indeed correlated with root development (Blilou et al., 2005; Vieten et al., 2005; Dello Ioio et al., 2007), affecting the formation, maintenance, and activity of RAM cells in deciduous trees (Palovaara and Hakman, 2009; Palovaara et al., 2010; Liu et al., 2014; Qi et al., 2020). PINs can significantly impact the rate of root growth and the size of the root tip (Vieten et al., 2005), possibly determining the pattern of taproot elongation in trees. Studies investigating the role of PINs in poplar (*Populus*), spruce (*Picea abies*), and pear (*Pyrus*), have reported a broader and more unique role for these proteins in auxin-controlled root development in trees (Palovaara et al., 2010; Liu et al., 2014; Qi et al., 2020). Some auxin-regulated developmental processes that are unique to woody plants (Liu et al., 2014), may directly affect the root apex expansion (also in taproots) toward wetter areas of the soil (van den Berg et al., 2016). Exploring the auxin regulatory network underlying root development will provide valuable information on the hormonal regulation of the formation and functioning of RAMs and the factors governing meristem size in plants with prominent taproots.

### Cytokinins

Cytokinins, as well as auxin, are required for the establishment and maintenance of RAM, through the enhanced of mitotic activity of quiescent center cells (QC; Zhang et al., 2013). In contrast to auxin, however, cytokinins control cell differentiation and inhibit root elongation. Studies on cytokinin biosynthesis mutants have shown that cytokinins can regulate the size of RAM. Application of exogenous cytokinins caused a decrease in meristem size, by affecting the rate of meristematic cell differentiation (Dello Ioio et al., 2007). In fact, a reduction in endogenous cytokinin levels in mutants (with a cytokinin level deficiency) results in faster growth of the primary root (Werner et al., 2001). Therefore, repression of cytokinin activity may enhance drought resistance in trees, enabling deeper soil exploitation by taproot elongation (Werner et al., 2001; Calvo-Polanco et al., 2019). Nevertheless, it is essential to determine if cytokinins function alone or interactively with other hormones do contribute to drought tolerance in plants.

### Ethylene

Ethylene, generating uneven transverse cell divisions in the QC of a RAM, plays a major role in inhibiting cell proliferation and root growth (Woeste et al., 1999; Schaller and Kieber, 2002; Růžička et al., 2007; Qin et al., 2019). An ethylene dependent pathway involved in inhibiting root elongation was identified in *ETHYLENE OVERPRODUCER* (*eto1*) mutants that exhibit enhanced ethylene biosynthesis, relative to wild-type plants, which produce long primary roots (Woeste et al., 1999). Higher root elongation in ethylene resistant *ETHYLENE RESISTANT 1* (*etr1*), *ETHYLENE INSENSITIVE2* (*ein2*), and *ETHYLENE INSENSITIVE3* (*ein3*) mutants also provided evidence that ethylene inhibits root growth (Růžička et al., 2007). The central function of ethylene in relation to root growth allows roots to restrict elongation when needed and extend their growth into deeper soil layers when conditions initiate growth restoration (Negi et al., 2010; Pandey et al., 2021). A lack of alterations in the size of root meristems in these ethylene mutants is consistent with the potential ability of certain taproots to first hold back and then restart growth under specific environmental conditions (Street et al., 2016). The ability to regulate cell elongation through ethylene, cytokinin, and auxin cross-talk may represent an efficient mechanism for directing the position of roots and may also be involved in plant response to drought conditions.

### Other Hormones

Gibberellins (GA), abscisic acid (ABA), and brassinosteroids (BR) are classes of hormones that can affect root development. Gibberellins act mainly on endodermal cells in root tissues, inducing an expansion of endodermal cells in the root elongation zone, which consequently limits the elongation rate of other root tissues (Ubeda-Tomás et al., 2008). The effect of ABA on root development has been shown to be concentration-dependent: low concentrations of ABA stimulate root elongation while higher concentration deters root formation (Harris, 2015; Rowe et al., 2016; Sun et al., 2020). Low concentrations of ABA enhance the activity of meristematic cells (stem cells) and alter auxin transport and signaling, while the suppressive effect of high concentrations of ABA on root growth are related to its inhibition of cell division in RAMs, as well as cells in the elongation zone (Sun et al., 2020).

Although auxin and ABA affect different aspects of root growth, high levels of ABA reduce auxin levels, which results in root growth inhibition due to the induction of PLT TFs (Yang et al., 2014; Promchuea et al., 2017). Indeed, when the level of drought is too severe, elevated levels of ABA inhibit root growth, which is why ABA is referred to as the stress hormone (Nakashima and Yamaguchi-Shinozaki, 2013). Interestingly, transgenic poplar lines with ectopic expression of *abi1* (*abscisic acid insensitive1*) exhibit an ABA insensitive phenotype, allowing plants exposed to a short-term water shortage an induction of primary root elongation (Sharp et al., 2004). The signaling pathway involving ABA interactions with ethylene, inhibits further primary root growth by increasing ethylene biosynthesis (Sharp et al., 2000; Qin et al., 2019). This suggests that the sensing of low ABA concentrations during episodes of water limitation could promote taproot growth. BR also promote root growth especially during drought periods. The BR biosynthesis maxima in the elongation zone is accomplished by the accumulation of osmoprotectant metabolites, resulting in the elongation of lateral roots and enhancing water uptake (Bao et al., 2004; Fàbregas et al., 2018; Vukašinović et al., 2021). Although examining of a specific hormone has made it possible to understand the mechanism of single hormone biosynthesis, perception, and signaling, the regulation of root development is largely dependent on the interaction of different hormone pathways.

### Hormonal Cross-Talk

Dynamic root growth is a result of the interaction between hormones affecting biosynthesis, transport, inactivation, perception, signaling pathways and regulating development, maintenance, and RAM function. An increase in auxin levels contributes to lower cytokinins levels. In addition, an increase in the level of cytokinin inhibits the synthesis of auxin (Eklof et al., 1997; Nordstrom et al., 2004; Di Mambro et al., 2017). Cytokinins may also affect, polar auxin transport and the formation of a local auxin gradient during lateral root formation as well as the expression of genes involved in auxin transport (Laplaze et al., 2007; Kuderova et al., 2008). Similarly, root growth is inhibited by the balance between auxin and ethylene. In response to ethylene, auxin accumulates in RAM cells and inhibits cell elongation and cell differentiation, consequently regulating how different components of the root system develop (Casson and Lindsey, 2003). The regulatory role of this balance was demonstrated through the use of mutants in which the biosynthesis, transport, and perception of auxin was affected (Růžička et al., 2007; Stepanova et al., 2007). The inhibition of *PLETHORA (PLT)* expression by *AUXIN RESPONSE FACTOR (ARF)*, which negatively regulates *WUSCHEL RELATED HOMEOBOX 5 (WOX5)* transcripts – the driver of stem cell formation – leads to distal stem cell differentiation in RAM (Figure 2; Su et al., 2011). Thus, the molecular interaction between auxin, cytokinins and other hormones controlling meristem development may be applied to the explanation of taproot growth. The question is which combinations regulate root elongation in a similar manner, or if the result varies in taproot vs. lateral root growth. Therefore, to understand the control of taproot growth, there is a need to explore the molecular and genetic mechanisms that regulates root development, through expression and functional analyses.

## Genetic Factors Involved in Root Development

Root development, as well as the hormonal regulation, are controlled by specific genes or groups of genes categorized as composite factors (Sarkar et al., 2007; Mitsis et al., 2020). Composite factor are induced when roots begin to grow, penetrating the soil, and determine both the growth of individual roots, as well as the overall architecture of the entire root system (Wachsman et al., 2015). Therefore, targeting the activation or suppression of gene expression is a key aspect of the genetic regulation of roots (Atkinson and Halfon, 2014). The genes encoding key TFs, hormone precursors and regulatory proteins collectively affect the functioning of the taproot. Moreover, they may act differently depending on the species. Elucidating the molecular mechanisms by which specific genes control the development of taproot’s RAM throughout a perennial lifetime, will provide valuable knowledge on every stage of root growth and aspect of root function (Slovak et al., 2016).

### Transcription Factors Involved in Root Development

The establishment of RAM is determined by many factors, including hormone levels, intercellular signaling, and receptors that interact with specific TFs activated in response to internal and external signals (Drisch and Stahl, 2015). Indeed, TFs in plants regulate the transcription of specific genes (Table 1), as well as the responses to external and internal stimuli (Mitsis et al., 2020). For example, MP-dependent TFs regulate auxin transport into cells and play a role in the generation of RAMs, and may control other auxin response genes (Weijers et al., 2006). TFs also play an important role in establishing the QC in embryonic roots and maintaining the QC in mature roots (Forzani et al., 2014). Establishing the QC is accomplished by determining the cell organization required for columella cell identity, and maintaining the undifferentiated status of the QC, which allows the QC to activate root growth to explore new soil spaces, increase root biomass, and enhance water absorption (Motte et al., 2019). Maintaining an area of undifferentiated stem cells in the RAM provides a source of cells needed to produce new roots throughout the lifetime of plants (Sarkar et al., 2007; Drisch and Stahl, 2015).

**TABLE 1:** The key genetic factors involved in root development.

Name	Abbr.	Family	Encodes	Functions	References
MONOPTEROS	MP	ARF	Transcription factor	root meristem establishment, pattern formation	Berleth and Jurgens, 1993
BODENLOS	BDL	AUX/IAA	Aux/IAA protein (IAA12)	root meristem establishment	Hamann et al., 2002
TARGET OF MONOPTEROS	TMO	bHLH	AP2 type transcription factor	root meristem establishment	Schlereth et al., 2010
WUSCHEL-RELATED HOMEOBOX5	WOX5	ATHB	Transcription factor	the columella stem cell identity	Sarkar et al., 2007
WUSCHEL-RELATED HOMEOBOX11	WOX11	ATHB	Transcription factor	meristem initiation, meristem maintenance and lateral root initiation	Hu and Xu, 2016
SCARECROW	SCR	GRAS	Transcription factor	maintaining the QC identity	Scheres et al., 1995
SHORTROOT	SHR	GRAS	Transcription factor	maintaining the QC identity	DiLaurenzio et al., 1996
PLETHORA	PLT	AP2/ERF	Transcription factor	maintaining the QC identity	Aida et al., 2004
ALTERED PHLOEM DEVELOPMENT	APL	MYB	MYB coiled-coil-type transcription factor	phloem identity	Bonke et al., 2003
III HOMEODOMAIN-LEUCINE ZIPPER	HD-ZIP III	HOMEODOMAIN-LEUCINE ZIPPER	Transcription factor	xylem tissues development	Carlsbecker et al., 2010

The ability of taproots to grow deeper may be associated with the maintenance of the columella stem cells in the distal meristem of root tip and regulation of auxin distribution as in lateral roots (Savina et al., 2020). Engaged in the above processes, WOX TFs (WOX 5/7 and WOX11) play a key role in inducing and sustaining primary roots growth, as well as generations of lateral roots, from a primary root (Hu and Xu, 2016; Baesso et al., 2018). For example, in poplar trees, the WOX TFs, WOX 4/5/11 and 12, regulate the development of new lateral roots originating from taproot (Baesso et al., 2020). Tree root systems can extend to considerable widths and depths, thus WOX increasing the ability of a tree to adapt to adverse abiotic and biotic conditions, such as drought or mechanical damage, to which they are exposed continuously. Indeed specific TFs associations have profound effects on plant resistance to drought e.g., the formation of root non-hair cells (Schiefelbein et al., 2014), the differentiation of root epidermal trichoblasts into root hair cells (Clowes, 2000; Ishida et al., 2008), as well as determining the root hair morphology (Bruex et al., 2012). The importance of TFs and the genes they regulate in taproot response under water deficit conditions, however, has not been investigated, and the specific role of TFs in enhancing drought resistance by promoting taproot growth, driven by ABA-regulated auxin transport, remains to be determined (Carlsbecker et al., 2010; Müller et al., 2016).

### Role of Micro RNA in the Regulation of Root Growth and Development

**FIGURE 3:**
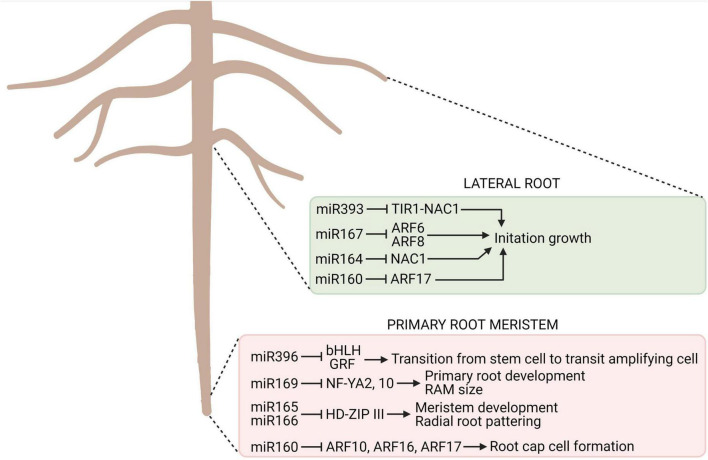
The key miRNAs involved in root development. For each type of structure, the implicated miRNA, their targets, and the process they control are indicated in the colored boxes. TIR, TRANSPORT INHIBITOR RESPONSE; ARF, AUXIN RESPONSE FACTOR; NAC, NO APICAL MERISTEM, ARABIDOPSIS TRANSCRIPTION ACTIVATION FACTOR (NAM, ATAF, CUC); bHLH, helix-loop-helix; GRF, GROWTH REGULATING FACTOR; HD-ZIP, HOMEODOMAIN LEUCINE ZIPPER; NF-YA, NUCLEAR FACTOR-YA [based on Couzigou and Combier (2016)]. Created with BioRender.com.

MicroRNAs (miRNA), along with other growth regulators, form networks controlling gene expressions at a developmental and tissue level, being key for the regulation of root development (Jones-Rhoades et al., 2006; Couzigou and Combier, 2016), also in deciduous and coniferous trees such as *Pinus tabuliformis*, *Larix olgensis*, and *Poncirus trifoliate* (Song et al., 2009; Zhang et al., 2013, 2019; Niu et al., 2015). Particularly, miRNAs play an important role in root morphogenesis, contributing to the regulation of meristem establishment and maintenance, vasculature differentiation, lateral and adventitious root formation, and the regulation of symbiotic interactions (Couzigou and Combier, 2016). The multitude of functional roles played by miRNAs, both in model, annual, and perennial plant species, confirms their integral role in root development (Figure 3). Little is known, however, about the role of miRNAs in the development of taproots in trees. Thus, understanding the role of these RNAs and their interactions with other molecular components, such as genes, TFs, and plant hormones, will assist in the elucidation of the complex pathways that control taproot development and function during foraging for water and nutrients, as overexpression of specific miRNAs increase tolerance to many abiotic stresses by changing root architecture and its adaptive responses to stressful conditions (Zhang, 2015). MicroRNAs and their interactions with other molecular components effectively regulate RAM size and the differentiation of vascular tissue in root, thus, represent a mechanism that could be applied to taproots growth (Khan et al., 2011). A comparison of PHV (PHAVOLUTA) and PHB (PHABULOSA) gene expression in long and short growing roots in miR165/166-resistant mutants indicated that these mutants have a reduced RAM size and a lower level of vascular differentiation than wild-type plants. Hence, miR165/166 regulates root development by controlling RAM size, organ polarity, differentiation of vascular elements, and shape of the root system architecture (Carlsbecker et al., 2010; Couzigou and Combier, 2016).

Hormone signal transduction pathways are also affected by miRNAs. For example, miR390 mediates the *miR390–TAS3–AUXIN RESPONSE FACTOR 2/ARF3/ARF4* regulatory pathway, which is involved in auxin signaling, and miR393 represses auxin signaling mediated by its downstream F-box auxin receptor targets, namely, TRANSPORT INHIBITOR RESPONSE 1 (TIR1), as well as AUXIN SIGNALING F-BOX PROTEINS 2 (AFB2) and AFB3 (Yoon et al., 2010; Meng et al., 2011). A negative regulation of ARF TFs by miR160 contributes to the maintenance of adequate auxin homeostasis and further lateral root formation (Wang et al., 2005; Meng et al., 2011), for example. Mutants resistant to miR160, however, exhibited reduced root branching (Couzigou and Combier, 2016). Another miRNA, miR390, expressed in cells located in the region of lateral root initiation downregulates *ARF2*, *ARF3*, and *ARF4*, resulting in the inhibition of lateral root growth (Marin et al., 2010). Furthermore, miR164 acts on the NAC1 TF acts downstream of TIR1 transmitting auxin signals, promotes lateral root emergence and controls lateral root elongation (miR167 acts on *ARF7* and *ARF19*) (Xie et al., 2004; Guo et al., 2005). The modulation of both the primary root and the lateral roots by miRNAs reveals the broad spectrum of action of these growth regulators in root development and function (Gutierrez et al., 2009).

The regulatory function of miRNAs may also affect drought resistance in roots enabling through the expression of drought-responsive genes. In this regard, some miRNAs, such as ABA responsive genes, auxin signaling genes, genes encoding osmolytes, and antioxidant defense-related genes, can promote an accumulation of target mRNAs associated with enhanced stress tolerance (Ding et al., 2013). Notably, many of the miRNAs that respond to drought stress have only been identified in trees such as poplar and larch, and have not been detected in annual plants, such as Arabidopsis or rice. This may indicate a specific role for miRNA in woody plant species with long-term root systems, whether they are broadleaf or coniferous tress. Accordingly, the ability of miRNAs to regulate gene expression in response to drought, may facilitate tree growth and survival under adverse conditions on a long-term basis (Osakabe et al., 2014). The regulation of both, lateral and primary roots growth (Gutierrez et al., 2009), increases the ability to explore of deeper soil layers. Nevertheless, our understanding of the mechanisms and genes controlling taproot growth, development, differentiation, function, and architecture, especially in response to adverse conditions, such as drought, is far from complete.

## Conclusion

The interaction of external and internal factors influences the growth and physiology of the taproot. The tips of a taproot consist of meristematic cells in the RAM. Assuming that the RAM is the main regulatory center responsible for taproot growth and cessation, a better understanding of the factors regulating the function of the RAM in taproots will provide fundamental information on the mechanisms that influence the development of the taproot. It is therefore necessary to understand the interactions between internal factors in the regulation of taproot growth and development, and to determine how these factors are related to external factors, e.g., drought. This raises the question of whether water restriction regulates and/or induces root growth in plants not only to maintain but also to accelerate root growth into deeper soil layers in response to water stress, and what internal factors are responsible for taproot development under drought stress. However, it is difficult to determine which one of these factors has a dominant effect on root growth, because the paths of dependence between external and internal factors are closely related and dependent on each other.

In the long term, understanding the regulatory role of genes, hormones, and microRNAs will help to improve the quality of nursery seedling production, including the development of effective management strategies that will allow the restoration of taproots in container cuttings. Unfortunately, the selection of specific strategies to improve the elongation of taproots in tree seedlings is challenging due to the variability of the reactions of roots to multiple internal and external influences. Under changing climate, manifested by high temperatures and reduced precipitation, the formation of a deep root system is crucial for the survival of seedlings, saplings and maturing tree.

## Author Contributions

PK drafted the manuscript. MZ sought funding for it. All authors contributed to the article review and editing, and approved the submitted version.

## Conflict of Interest

The authors declare that the research was conducted in the absence of any commercial or financial relationships that could be construed as a potential conflict of interest.

## Publisher’s Note

All claims expressed in this article are solely those of the authors and do not necessarily represent those of their affiliated organizations, or those of the publisher, the editors and the reviewers. Any product that may be evaluated in this article, or claim that may be made by its manufacturer, is not guaranteed or endorsed by the publisher.

## References

[B1] Aida M. Beis D. Heidstra R. Willemsen V. Blilou I. Galinha C. (2004). The PLETHORA genes mediate patterning of the *Arabidopsis* root stem cell niche. *Cell* 119 109–120. 10.1016/j.cell.2004.09.018 15454085

[B2] Allen C. D. Breshears D. D. McDowell N. G. (2015). On underestimation of global vulnerability to tree mortality and forest die-off from hotter drought in the Anthropocene. *Ecosphere* 6:55. 10.1890/es15-00203.1

[B3] Arend M. Kuster T. Gunthardt-Goerg M. S. Dobbertin M. (2011). Provenance-specific growth responses to drought and air warming in three European oak species (*Quercus robur*, *Q.petraea* and *Q.pubescens*). *Tree Physiol.* 31 287–297. 10.1093/treephys/tpr004 21422189

[B4] Atkinson T. J. Halfon M. S. (2014). Regulation of gene expression in the genomic context. *Comp. Struct. Biotechnol. J.* 9:e201401001. 10.5936/csbj.201401001 24688749 PMC3962188

[B5] Baesso B. Chiatante D. Terzaghi M. Zenga D. Nieminen K. Mahonen A. P. (2018). Transcription factors PRE3 and WOX11 are involved in the formation of new lateral roots from secondary growth taproot in *A.thaliana*. *Plant Biol.* 20 426–432. 10.1111/plb.12711 29450949

[B6] Baesso B. Terzaghi M. Chiatante D. Scippa G. S. Montagnoli A. (2020). WOX genes expression during the formation of new lateral roots from secondary structures in *Populus nigra* (L.) taproot. *Sci. Rep.* 10:6. 10.1038/s41598-020-75150-1 33144589 PMC7641218

[B7] Baluška F. Mancuso S. Volkmann D. Barlow P. W. (2010). Root apex transition zone: a signalling-response nexus in the root. *Trends Plant Sci.* 15 402–408. 10.1016/j.tplants.2010.04.007 20621671

[B8] Bao F. Shen J. J. Brady S. R. Muday G. K. Asami T. Yang Z. B. (2004). Brassinosteroids interact with auxin to promote lateral root development in *Arabidopsis*. *Plant Physiol.* 134 1624–1631. 10.1104/pp.103.036897 15047895 PMC419836

[B9] Barbeta A. Peñuelas J. (2017). Relative contribution of groundwater to plant transpiration estimated with stable isotopes. *Sci. Rep.* 7:10. 10.1038/s41598-017-09643-x 28874685 PMC5585407

[B10] Beemster G. T. S. Baskin T. I. (2000). Mediates effects of cytokinin, but not of auxin, on cell division and expansion in the root of *Arabidopsis*. *Plant Physiol.* 124 1718–1727. 10.1104/pp.124.4.1718 11115888 PMC59869

[B11] Benková E. Hejatko J. (2009). Hormone interactions at the root apical meristem. *Plant Mol. Biol.* 69 383–396. 10.1007/s11103-008-9393-6 18807199

[B12] Berleth T. Jurgens G. (1993). The role of the monopteros gene in organizing the basal body region of the *Arabidopsis* embryo. *Development* 118 575–587.

[B13] Bleby T. M. McElrone A. J. Jackson R. B. (2010). Water uptake and hydraulic redistribution across large woody root systems to 20 m depth. *Plant Cell Environ.* 33 2132–2148. 10.1111/j.1365-3040.2010.02212.x 20716068

[B14] Blilou I. Xu J. Wildwater M. Willemsen V. Paponov I. Friml J. (2005). The PIN auxin efflux facilitator network controls growth and patterning in *Arabidopsis* roots. *Nature* 433 39–44. 10.1038/nature03184 15635403

[B15] Bonke M. Thitamadee S. Mähönen A. P. Hauser M. T. Helariutta Y. (2003). APL regulates vascular tissue identity in *Arabidopsis*. *Nature* 426 181–186. 10.1038/nature02100 14614507

[B16] Bréda N. Huc R. Granier A. Dreyer E. (2006). Temperate forest trees and stands under severe drought: a review of ecophysiological responses, adaptation processes and long-term consequences. *Ann. For. Sci.* 63 625–644. 10.1051/forest:2006042

[B17] Bruex A. Kainkaryam R. M. Wieckowski Y. Kang Y. H. Bernhardt C. Xia Y. (2012). A gene regulatory network for root epidermis cell differentiation in *Arabidopsis*. *PLoS Genet.* 8:20. 10.1371/journal.pgen.1002446 22253603 PMC3257299

[B18] Brunner I. Herzog C. Dawes M. A. Arend M. Sperisen C. (2015). How tree roots respond to drought. *Front. Plant Sci.* 6:16. 10.3389/fpls.2015.00547 26284083 PMC4518277

[B19] Calvo-Polanco M. Ruiz-Lozano J. M. Azcón R. Molina S. Beuzon C. R. García J. L. (2019). Phenotypic and molecular traits determine the tolerance of olive trees to drought stress. *Plant Physiol. Biochem.* 139 521–527. 10.1016/j.plaphy.2019.04.017 31015091

[B20] Carlsbecker A. Lee J. Y. Roberts C. J. Dettmer J. Lehesranta S. Zhou J. (2010). Cell signalling by microRNA165/6 directs gene dose-dependent root cell fate. *Nature* 465 316–321. 10.1038/nature08977 20410882 PMC2967782

[B21] Casson S. A. Lindsey K. (2003). Genes and signalling in root development. *New Phytol.* 158 11–38. 10.1046/j.1469-8137.2003.00705.x

[B22] Chapman K. Groot E. P. Nichol S. A. Rost T. L. (2002). Primary root growth and the pattern of root apical meristem organization are coupled. *J. Plant Growth Regul.* 21 287–295. 10.1007/s00344-002-0036-x

[B23] Choat B. Brodribb T. J. Brodersen C. R. Duursma R. A. López R. Medlyn B. E. (2018). Triggers of tree mortality under drought. *Nature* 558 531–539. 10.1038/s41586-018-0240-x 29950621

[B24] Clowes F. A. L. (2000). Pattern in root meristem development in angiosperms. *New Phytol.* 146 83–94. 10.1046/j.1469-8137.2000.00614.x

[B25] Couzigou J. M. Combier J. P. (2016). Plant microRNAs: key regulators of root architecture and biotic interactions. *New Phytol.* 212 22–35. 10.1111/nph.14058 27292927

[B26] David T. S. Pinto C. A. Nadezhdina N. Kurz-Besson C. Henriques M. O. Quilhó T. (2013). Root functioning, tree water use and hydraulic redistribution in *Quercus suber* trees: a modeling approach based on root sap flow. *For. Ecol. Manage.* 307 136–146. 10.1016/j.foreco.2013.07.012

[B27] Dello Ioio R. Linhares F. S. Scacchi E. Casamitjana-Martinez E. Heidstra R. Costantino P. (2007). Cytokinins determine *Arabidopsis* root-meristem size by controlling cell differentiation. *Curr. Biol.* 17 678–682. 10.1016/j.cub.2007.02.047 17363254

[B28] Di Iorio A. Lasserre B. Scippa G. S. Chiatante D. (2005). Root system architecture of *Quercus pubescens* trees growing on different sloping conditions. *Ann. Bot.* 95 351–361. 10.1093/aob/mci033 15567806 PMC4246836

[B29] Di Mambro R. De Ruvo M. Pacifici E. Salvi E. Sozzani R. Benfey P. N. (2017). Auxin minimum triggers the developmental switch from cell division to cell differentiation in the *Arabidopsis* root. *Proc. Natl. Acad. Sci. U.S.A.* 114 E7641–E7649. 10.1073/pnas.1705833114 28831001 PMC5594665

[B30] DiLaurenzio L. Wysocka-Diller J. Malamy J. E. Pysh L. Helariutta Y. Freshour G. (1996). The *SCARECROW* gene regulates an asymmetric cell division that is essential for generating the radial organization of the *Arabidopsis* root. *Cell* 86 423–433. 10.1016/s0092-8674(00)80115-4 8756724

[B31] Ding Y. F. Tao Y. L. Zhu C. (2013). Emerging roles of microRNAs in the mediation of drought stress response in plants. *J. Exp. Bot.* 64 3077–3086. 10.1093/jxb/ert164 23814278

[B32] Dolan L. Janmaat K. Willemsen V. Linstead P. Poethig S. Roberts K. (1993). Cellular-organization of the *Arabidopsis thaliana* root. *Development* 119 71–84. 8275865 10.1242/dev.119.1.71

[B33] Domec J. C. Warren J. M. Meinzer F. C. Brooks J. R. Coulombe R. (2004). Native root xylem embolism and stomatal closure in stands of Douglas-fir and ponderosa pine: mitigation by hydraulic redistribution. *Oecologia* 141 7–16. 10.1007/s00442-004-1621-4 15338263

[B34] Drisch R. C. Stahl Y. (2015). Function and regulation of transcription factors involved in root apical meristem and stem cell maintenance. *Front. Plant Sci.* 6:8. 10.3389/fpls.2015.00505 26217359 PMC4491714

[B35] Dubrovsky J. G. Gómez-Lomelí L. F. (2003). Water deficit accelerates determinate developmental program of the primary root and does not affect lateral root initiation in a Sonoran Desert cactus (*Pachycereus pringlei*. Cactaceae). *Am. J. Bot.* 90 823–831. 10.3732/ajb.90.6.823 21659177

[B36] Eklof S. Astot C. Blackwell J. Moritz T. Olsson O. Sandberg G. (1997). Auxin-cytokinin interactions in wild-type and transgenic tobacco. *Plant Cell Physiol.* 38 225–235. 10.1093/oxfordjournals.pcp.a029157

[B37] Fàbregas N. Lozano-Elena F. Blasco-Escámez D. Tohge T. Martínez-Andújar C. Albacete A. (2018). Overexpression of the vascular brassinosteroid receptor BRL3 confers drought resistance without penalizing plant growth. *Nat. Commun.* 9:13. 10.1038/s41467-018-06861-3 30409967 PMC6224425

[B38] Forzani C. Aichinger E. Sornay E. Willemsen V. Laux T. Dewitte W. (2014). WOX5 suppresses CYCLIN D activity to establish quiescence at the center of the root stem cell niche. *Curr. Biol.* 24 1939–1944. 10.1016/j.cub.2014.07.019 25127220 PMC4148176

[B39] Freschet G. T. Roumet C. Comas L. H. Weemstra M. Bengough A. G. Rewald B. (2021a). Root traits as drivers of plant and ecosystem functioning: current understanding, pitfalls and future research needs. *New Phytol.* 232 1123–1158. 10.1111/nph.17072 33159479

[B40] Freschet G. T. Pagès L. Iversen C. M. Comas L. H. Rewald B. Roumet C. (2021b). A starting guide to root ecology: strengthening ecological concepts and standardising root classification, sampling, processing and trait measurements. *New Phytol.* 232 973–1122. 10.1111/nph.17572 34608637 PMC8518129

[B41] Galinha C. Hofhuis H. Luijten M. Willemsen V. Blilou I. Heidstra R. (2007). PLETHORA proteins as dose-dependent master regulators of *Arabidopsis* root development. *Nature* 449 1053–1057. 10.1038/nature06206 17960244

[B42] Guo H. S. Xie Q. Fei J. F. Chua N. H. (2005). MicroRNA directs mRNA cleavage of the transcription factor NAC1 to downregulate auxin signals for *Arabidopsis* lateral root development. *Plant Cell* 17 1376–1386. 10.1105/tpc.105.030841 15829603 PMC1091761

[B43] Gupta A. Rico-Medina A. Caño-Delgado A. I. (2020). The physiology of plant responses to drought. *Science* 368 266–269. 10.1126/science.aaz7614 32299946

[B44] Gutierrez L. Bussell J. D. Păcurar D. I. Schwambach J. Păcurar M. Bellini C. (2009). Phenotypic plasticity of adventitious rooting in *Arabidopsis* is controlled by complex regulation of AUXIN RESPONSE FACTOR transcripts and microrna abundance. *Plant Cell* 21 3119–3132. 10.1105/tpc.108.064758 19820192 PMC2782293

[B45] Hamann T. Benková E. Bäurle I. Kientz M. Jürgens G. (2002). The *Arabidopsis BODENLOS* gene encodes an auxin response protein inhibiting MONOPTEROS-mediated embryo patterning. *Genes Dev.* 16 1610–1615. 10.1101/gad.229402 12101120 PMC186366

[B46] Harris J. M. (2015). Abscisic acid: hidden architect of root system structure. *Plants-Basel* 4 548–572. 10.3390/plants4030548 27135341 PMC4844405

[B47] Hu X. Xu L. (2016). Transcription factors WOX11/12 directly activate *WOX5/7* to promote root primordia initiation and organogenesis. *Plant Physiol.* 172 2363–2373. 10.1104/pp.16.01067 27784768 PMC5129711

[B48] Ishida T. Adachi S. Yoshimura M. Shimizu K. Umeda M. Sugimoto K. (2010). Auxin modulates the transition from the mitotic cycle to the endocycle in *Arabidopsis*. *Development* 137 63–71. 10.1242/dev.035840 20023161

[B49] Ishida T. Kurata T. Okada K. Wada T. (2008). A genetic regulatory network in the development of trichomes and root hairs. *Annu. Rev. Plant Biol.* 59 365–386. 10.1146/annurev.arplant.59.032607.092949 18257710

[B50] Jiang K. Feldman L. J. (2010). Positioning of the auxin maximum affects the character of cells occupying the root stem cell niche. *Plant Signal. Behav.* 5 202–204. 10.4161/psb.5.2.11188 20173411 PMC2884136

[B51] Jones-Rhoades M. W. Bartel D. P. Bartel B. (2006). MicroRNAs and their regulatory roles in plants. *Annu. Rev. Plant Biol.* 57 19–53. 10.1146/annurev.arplant.57.032905.105218 16669754

[B52] Jung J. K. H. McCouch S. (2013). Getting to the roots of it: genetic and hormonal control of root architecture. *Front. Plant Sci.* 4:32. 10.3389/fpls.2013.00186 23785372 PMC3685011

[B53] Khan G. A. Declerck M. Sorin C. Hartmann C. Crespi M. Lelandais-Brière C. (2011). MicroRNAs as regulators of root development and architecture. *Plant Mol. Biol.* 77 47–58. 10.1007/s11103-011-9793-x 21607657

[B54] Kuderova A. Urbankova I. Valkova M. Malbeck J. Brzobohaty B. Nemethova D. (2008). Effects of conditional IPT-Dependent cytokinin overproduction on root architecture of *Arabidopsis* seedlings. *Plant Cell Physiol.* 49 570–582. 10.1093/pcp/pcn029 18296451

[B55] Laplaze L. Benková E. Casimiro I. Maes L. Vanneste S. Swarup R. (2007). Cytokinins act directly on lateral root founder cells to inhibit root initiation. *Plant Cell* 19 3889–3900. 10.1105/tpc.107.055863 18065686 PMC2217640

[B56] Liu J. C. Sheng L. H. Xu Y. Q. Li J. Q. Yang Z. N. Huang H. (2014). WOX11 and *12* are involved in the first-step cell fate transition during de novo root organogenesis in *Arabidopsis*. *Plant Cell* 26 1081–1093. 10.1105/tpc.114.122887 24642937 PMC4001370

[B57] Löf M. Welander N. T. (2004). Influence of herbaceous competitors on early growth in direct seeded *Fagus sylvatica* L. and *Quercus robur* L. *Ann. For. Sci.* 61 781–788. 10.1051/forest:2004075

[B58] Lynch J. Marschner P. Rengel Z. (2012). *Effect of Internal and External Factors on Root Growth and Development.* San Diego, CA: Elsevier Academic Press Inc.

[B59] Mackay D. S. Savoy P. R. Grossiord C. Tai X. N. Pleban J. R. Wang D. R. (2020). Conifers depend on established roots during drought: results from a coupled model of carbon allocation and hydraulics. *New Phytol.* 225 679–692. 10.1111/nph.16043 31276231

[B60] Marin E. Jouannet V. Herz A. Lokerse A. S. Weijers D. Vaucheret H. (2010). miR390, *Arabidopsis* TAS3 tasiRNAs, and their AUXIN RESPONSE factor targets define an autoregulatory network quantitatively regulating lateral root growth. *Plant Cell* 22 1104–1117. 10.1105/tpc.109.072553 20363771 PMC2879756

[B61] McCormack M. L. Dickie I. A. Eissenstat D. M. Fahey T. J. Fernandez C. W. Guo D. L. (2015). Redefining fine roots improves understanding of below-ground contributions to terrestrial biosphere processes. *New Phytol.* 207 505–518. 10.1111/nph.13363 25756288

[B62] McCormack M. L. Guo D. L. Iversen C. M. Chen W. L. Eissenstat D. M. Fernandez C. W. (2017). Building a better foundation: improving root-trait measurements to understand and model plant and ecosystem processes. *New Phytol.* 215 27–37. 10.1111/nph.14459 28295373

[B63] Meng Y. Shao C. Wang H. Chen M. (2011). The regulatory activities of plant microRNAs: a more dynamic perspective. *Plant Physiol.* 157 1583–1595. 10.1104/pp.111.187088 22003084 PMC3327222

[B64] Mira M. M. Huang S. L. Kapoor K. Hammond C. Hill R. D. Stasolla C. (2017). Expression of *Arabidopsis* class 1 phytoglobin (AtPgb1) delays death and degradation of the root apical meristem during severe PEG-induced water deficit. *J. Exp. Bot.* 68 5653–5668. 10.1093/jxb/erx371 29059380 PMC5853930

[B65] Mitsis T. Efthimiadou A. Bacopoulou F. Vlachakis D. Chrousos G. P. Eliopoulos E. (2020). Transcription factors and evolution: an integral part of gene expression (Review). *World Acad. Sci. J.* 2 3–8. 10.3892/wasj.2020.32

[B66] Montagnoli A. Dumroese R. K. Terzaghi M. Onelli E. Scippa G. S. Chiatante D. (2019). Seasonality of fine root dynamics and activity of root and shoot vascular cambium in a *Quercus ilex L*. forest (Italy). *For. Ecol. Manage.* 431 26–34. 10.1016/j.foreco.2018.06.044

[B67] Motte H. Vanneste S. Beeckman T. (2019). “Molecular and environmental regulation of root development,” in *Annual Review of Plant Biology*, Vol. 70 ed. Merchant S. S. (Palo Alto, CA: Annual Reviews), 465–488. 10.1146/annurev-arplant-050718-100423 30822115

[B68] Mucha J. Jagodziński A. M. Bułaj B. Łakomy P. Talaśka A. M. Oleksyn J. (2018). Functional response of *Quercus robur* L. to taproot pruning: a 5-year case study. *Ann. For. Sci.* 75:12. 10.1007/s13595-018-0708-8

[B69] Müller C. J. Valdés A. E. Wang G. Ramachandran P. Beste L. Uddenberg D. (2016). PHABULOSA mediates an auxin signaling loop to regulate vascular patterning in *Arabidopsis*. *Plant Physiol.* 170 956–970. 10.1104/pp.15.01204 26637548 PMC4734557

[B70] Nakashima K. Yamaguchi-Shinozaki K. (2013). ABA signaling in stress-response and seed development. *Plant Cell Rep.* 32 959–970. 10.1007/s00299-013-1418-1 23535869

[B71] Negi S. Sukumar P. Liu X. Cohen J. D. Muday G. K. (2010). Genetic dissection of the role of ethylene in regulating auxin-dependent lateral and adventitious root formation in tomato. *Plant J.* 61 3–15. 10.1111/j.1365-313X.2009.04027.x 19793078

[B72] Niu S. H. Liu C. Yuan H. W. Li P. Li W. (2015). Identification and expression profiles of sRNAs and their biogenesis and action-related genes in male and female cones of *Pinus tabuliformis*. *BMC Genomics* 16:13. 10.1186/s12864-015-1885-6 26369937 PMC4570457

[B73] Nordstrom A. Tarkowski P. Tarkowska D. Norbaek R. Astot C. Dolezal K. (2004). Auxin regulation of cytokinin biosynthesis in *Arabidopsis thaliana*: a factor of potential importance for auxin-cytokinin-regulated development. *Proc. Natl. Acad. Sci. U.S.A.* 101 8039–8044. 10.1073/pnas.0402504101 15146070 PMC419553

[B74] Osakabe Y. Osakabe K. Shinozaki K. Tran L. S. (2014). Response of plants to water stress. *Front. Plant Sci.* 5:86. 10.3389/fpls.2014.00086 24659993 PMC3952189

[B75] Osonubi O. Davies W. J. (1981). Root-growth and water relations of oak and birch seedlings. *Oecologia* 51 343–350. 10.1007/bf00540904 28310018

[B76] Palovaara J. Hakman I. (2009). *WOX2* and polar auxin transport during spruce embryo pattern formation. *Plant Signal. Behav.* 4 153–155. 10.4161/psb.4.2.7684 19649198 PMC2637508

[B77] Palovaara J. Hallberg H. Stasolla C. Hakman I. (2010). Comparative expression pattern analysis of *WUSCHEL-related homeobox 2* (*WOX2*) and *WOX8/9* in developing seeds and somatic embryos of the gymnosperm *Picea abies*. *New Phytol.* 188 122–135. 10.1111/j.1469-8137.2010.03336.x 20561212

[B78] Pandey B. K. Huang G. Q. Bhosale R. Hartman S. Sturrock C. J. Jose L. (2021). Plant roots sense soil compaction through restricted ethylene diffusion. *Science* 371 276–280. 10.1126/science.abf3013 33446554

[B79] Perilli S. Di Mambro R. Sabatini S. (2012). Growth and development of the root apical meristem. *Curr. Opin. Plant Biol.* 15 17–23. 10.1016/j.pbi.2011.10.006 22079783

[B80] Peters W. S. Tomos A. D. (1996). The history of tissue tension. *Ann. Bot.* 77 657–665. 10.1006/anbo.1996.0082 11541099

[B81] Petricka J. J. Winter C. M. Benfey P. N. (2012). Control of *Arabidopsis* root development. *Annu. Rev. Plant Biol.* 63 563–590. 10.1146/annurev-arplant-042811-105501 22404466 PMC3646660

[B82] Promchuea S. Zhu Y. J. Chen Z. Z. Zhang J. Gong Z. Z. (2017). ARF2 coordinates with PLETHORAs and PINs to orchestrate ABA-mediated root meristem activity in *Arabidopsis*. *J. Integr. Plant Biol.* 59 30–43. 10.1111/jipb.12506 28074634

[B83] Qi L. Y. Chen L. Wang C. S. Zhang S. L. Yang Y. J. Liu J. L. (2020). Characterization of the auxin efflux transporter PIN proteins in pear. *Plants-Basel* 9:17. 10.3390/plants9030349 32164258 PMC7154836

[B84] Qin H. He L. N. Huang R. F. (2019). The coordination of ethylene and other hormones in primary root development. *Front. Plant Sci.* 10:8. 10.3389/fpls.2019.00874 31354757 PMC6635467

[B85] Rahman A. Bannigan A. Sulaman W. Pechter P. Blancaflor E. B. Baskin T. I. (2007). Auxin, actin and growth of the *Arabidopsis thaliana* primary root. *Plant J.* 50 514–528. 10.1111/j.1365-313X.2007.03068.x 17419848

[B86] Ristova D. Giovannetti M. Metesch K. Busch W. (2018). Natural genetic variation shapes root system responses to phytohormones in *Arabidopsis*. *Plant J.* 96 468–481. 10.1111/tpj.14034 30030851 PMC6220887

[B87] Robbins N. E. Dinneny J. R. (2018). Growth is required for perception of water availability to pattern root branches in plants. *Proc. Natl. Acad. Sci. U.S.A.* 115 E822–E831. 10.1073/pnas.1710709115 29317538 PMC5789911

[B88] Rowe J. H. Topping J. F. Liu J. L. Lindsey K. (2016). Abscisic acid regulates root growth under osmotic stress conditions via an interacting hormonal network with cytokinin, ethylene and auxin. *New Phytol.* 211 225–239. 10.1111/nph.13882 26889752 PMC4982081

[B89] Růžička K. Ljung K. Vanneste S. Podhorská R. Beeckman T. Friml J. (2007). Ethylene regulates root growth through effects on auxin biosynthesis and transport-dependent auxin distribution. *Plant Cell* 19 2197–2212. 10.1105/tpc.107.052126 17630274 PMC1955700

[B90] Sabatini S. Heidstra R. Wildwater M. Scheres B. (2003). SCARECROW is involved in positioning the stem cell niche in the *Arabidopsis* root meristem. *Genes Dev.* 17 354–358. 10.1101/gad.252503 12569126 PMC195985

[B91] Sarkar A. K. Luijten M. Miyashima S. Lenhard M. Hashimoto T. Nakajima K. (2007). Conserved factors regulate signalling in *Arabidopsis thaliana* shoot and root stem cell organizers. *Nature* 446 811–814. 10.1038/nature05703 17429400

[B92] Savina M. S. Pasternak T. Omelyanchuk N. A. Novikova D. D. Palme K. Mironova V. V. (2020). Cell dynamics in WOX5-overexpressing root tips: the impact of local auxin biosynthesis. *Front. Plant Sci.* 11:13. 10.3389/fpls.2020.560169 33193486 PMC7642516

[B93] Schaller G. E. Kieber J. J. (2002). Ethylene. *Arabidopsis Book* 1:e0071. 10.1199/tab.0071 22303216 PMC3243340

[B94] Scheres B. Dilaurenzio L. Willemsen V. Hauser M. T. Janmaat K. Weisbeek P. (1995). Mutations affecting the radial organization of the *Arabidopsis* root display specific defects throughout the embryonic axis. *Development* 121 53–62.

[B95] Schiefelbein J. Huang L. Zheng X. H. (2014). Regulation of epidermal cell fate in *Arabidopsis* roots: the importance of multiple feedback loops. *Front. Plant Sci.* 5:4. 10.3389/fpls.2014.00047 24596575 PMC3925829

[B96] Schlereth A. Möller B. Liu W. L. Kientz M. Flipse J. Rademacher E. H. (2010). MONOPTEROS controls embryonic root initiation by regulating a mobile transcription factor. *Nature* 464 913–U128. 10.1038/nature08836 20220754

[B97] Sharp R. E. LeNoble M. E. Else M. A. Thorne E. T. Gherardi F. (2000). Endogenous ABA maintains shoot growth in tomato independently of effects on plant water balance: evidence for an interaction with ethylene. *J. Exp. Bot.* 51 1575–1584. 10.1093/jexbot/51.350.1575 11006308

[B98] Sharp R. E. Poroyko V. Hejlek L. G. Spollen W. G. Springer G. K. Bohnert H. J. (2004). Root growth maintenance during water deficits: physiology to functional genomics. *J. Exp. Bot.* 55 2343–2351. 10.1093/jxb/erh276 15448181

[B99] Shishkova S. Las Peñas M. L. Napsucialy-Mendivil S. Matvienko M. Kozik A. Montiel J. (2013). Determinate primary root growth as an adaptation to aridity in Cactaceae: towards an understanding of the evolution and genetic control of the trait. *Ann. Bot.* 112 239–252. 10.1093/aob/mct100 23666887 PMC3698391

[B100] Shishkova S. Rost T. L. Dubrovsky J. G. (2008). Determinate root growth and meristem maintenance in angiosperms. *Ann. Bot.* 101 319–340. 10.1093/aob/mcm251 17954472 PMC2701811

[B101] Skiadaresis G. Schwarz J. A. Bauhus J. (2019). Groundwater extraction in floodplain forests reduces radial growth and increases summer drought sensitivity of pedunculate oak trees (*Quercus robur* L.). *Front. For. Glob. Change* 2:16. 10.3389/ffgc.2019.00005

[B102] Slovak R. Ogura T. Satbhai S. B. Ristova D. Busch W. (2016). Genetic control of root growth: from genes to networks. *Ann. Bot.* 117 9–24. 10.1093/aob/mcv160 26558398 PMC4701154

[B103] Smart D. R. Carlisle E. Goebel M. Núñez B. A. (2005). Transverse hydraulic redistribution by a grapevine. *Plant Cell Environ.* 28 157–166. 10.1111/j.1365-3040.2004.01254.x

[B104] Song C. N. Fang J. G. Li X. Y. Liu H. Chao C. T. (2009). Identification and characterization of 27 conserved microRNAs in citrus. *Planta* 230 671–685. 10.1007/s00425-009-0971-x 19585144 PMC2729984

[B105] Sozzani R. Iyer-Pascuzzi A. (2014). Postembryonic control of root meristem growth and development. *Curr. Opin. Plant Biol.* 17 7–12. 10.1016/j.pbi.2013.10.005 24507488

[B106] Stepanova A. N. Robertson-Hoyt J. Yun J. Benavente L. M. Xie D. Y. DoleZal K. (2008). TAA1-mediated auxin biosynthesis is essential for hormone crosstalk and plant development. *Cell* 133 177–191. 10.1016/j.cell.2008.01.047 18394997

[B107] Stepanova A. N. Yun J. Likhacheva A. V. Alonso J. M. (2007). Multilevel interactions between ethylene and auxin in *Arabidopsis* roots. *Plant Cell* 19 2169–2185. 10.1105/tpc.107.052068 17630276 PMC1955696

[B108] Street I. H. Mathews D. E. Yamburkenko M. V. Sorooshzadeh A. John R. T. Swarup R. (2016). Cytokinin acts through the auxin influx carrier AUX1 to regulate cell elongation in the root. *Development* 143 3982–3993. 10.1242/dev.132035 27697901 PMC5117139

[B109] Su Y. H. Liu Y. B. Zhang X. S. (2011). Auxin-cytokinin interaction regulates meristem development. *Mol. Plant* 4 616–625. 10.1093/mp/ssr007 21357646 PMC3146736

[B110] Sun Y. F. Pri-Tal O. Michaeli D. Mosquna A. (2020). Evolution of abscisic acid signaling module and its perception. *Front. Plant Sci.* 11:9. 10.3389/fpls.2020.00934 32754170 PMC7367143

[B111] Tuberosa R. (2012). Phenotyping for drought tolerance of crops in the genomics era. *Front. Physiol.* 3:26. 10.3389/fphys.2012.00347 23049510 PMC3446691

[B112] Ubeda-Tomás S. Swarup R. Coates J. Swarup K. Laplaze L. Beemster G. T. S. (2008). Root growth in *Arabidopsis* requires gibberellin/DELLA signalling in the endodermis. *Nat. Cell Biol.* 10 625–628. 10.1038/ncb1726 18425113

[B113] van den Berg T. Korver R. A. Testerink C. ten Tusscher K. (2016). Modeling halotropism: a key role for root tip architecture and reflux loop remodeling in redistributing auxin. *Development* 143 3350–3362. 10.1242/dev.135111 27510970 PMC5047658

[B114] Vieten A. Vanneste S. Wisniewska J. Benková E. Benjamins R. Beeckman T. (2005). Functional redundancy of PIN proteins is accompanied by auxindependent cross-regulation of PIN expression. *Development* 132 4521–4531. 10.1242/dev.02027 16192309

[B115] Vukašinović N. Wang Y. W. Vanhoutte I. Fendrych M. Guo B. Y. Kvasnica M. (2021). Local brassinosteroid biosynthesis enables optimal root growth. *Nat. Plants* 7 619–632. 10.1038/s41477-021-00917-x 34007032

[B116] Wachsman G. Sparks E. E. Benfey P. N. (2015). Genes and networks regulating root anatomy and architecture. *New Phytol.* 208 26–38. 10.1111/nph.13469 25989832

[B117] Wang J. W. Wang L. J. Mao Y. B. Cai W. J. Xue H. W. Chen X. Y. (2005). Control of root cap formation by microRNA-targeted auxin response factors in *Arabidopsis*. *Plant Cell* 17 2204–2216. 10.1105/tpc.105.033076 16006581 PMC1182483

[B118] Wang L. K. Chu H. W. Li Z. Y. Wang J. Li J. T. Qiao Y. (2014). Origin and development of the root cap in rice. *Plant Physiol.* 166 603–U223. 10.1104/pp.114.240929 24958716 PMC4213092

[B119] Weijers D. Schlereth A. Ehrismann J. S. Schwank G. Kientz M. Jürgens G. (2006). Auxin triggers transient local signaling for cell specification in *Arabidopsis* embryogenesis. *Dev. Cell* 10 265–270. 10.1016/j.devcel.2005.12.001 16459305

[B120] Werner T. Motyka V. Strnad M. Schmülling T. (2001). Regulation of plant growth by cytokinin. *Proc. Natl. Acad. Sci. U.S.A.* 98 10487–10492. 10.1073/pnas.171304098 11504909 PMC56987

[B121] Woeste K. E. Ye C. Kieber J. J. (1999). Two *Arabidopsis* mutants that overproduce ethylene are affected in the posttranscriptional regulation of 1-aminocyclopropane-1-carboxylic acid synthase. *Plant Physiol.* 119 521–530. 10.1104/pp.119.2.521 9952448 PMC32129

[B122] Xie Z. X. Johansen L. K. Gustafson A. M. Kasschau K. D. Lellis A. D. Zilberman D. (2004). Genetic and functional diversification of small RNA pathways in plants. *PLoS Biol.* 2:E104. 10.1371/journal.pbio.0020104 15024409 PMC350667

[B123] Xuan W. Band L. R. Kumpf R. P. Van Damme D. Parizot B. De Rop G. (2016). Cyclic programmed cell death stimulates hormone signaling and root development in *Arabidopsis*. *Science* 351 384–387. 10.1126/science.aad2776 26798015

[B124] Yang L. Zhang J. He J. N. Qin Y. Y. Hua D. P. Duan Y. (2014). ABA-mediated ROS in mitochondria regulate root meristem activity by controlling *PLETHORA* expression in *Arabidopsis*. *PLoS Genet.* 10:18. 10.1371/journal.pgen.1004791 25522358 PMC4270459

[B125] Yoon E. K. Yang J. H. Lee W. S. (2010). Auxin and abscisic acid responses of auxin response factor 3 in *Arabidopsis* lateral root development. *J. Plant Biol.* 53 150–154. 10.1007/s12374-010-9100-4

[B126] Zadworny M. Jagodziński A. M. Łakomy P. Mucha J. Oleksyn J. Rodríguez-Calcerrada J. (2019). Regeneration origin affects radial growth patterns preceding oak decline and death - insights from tree-ring δC-13 and δO-18. *Agricult. Forest Meteorol.* 278:12. 10.1016/j.agrformet.2019.107685

[B127] Zadworny M. Jagodziński A. M. Łakomy P. Ufnalski K. Oleksyn J. (2014). The silent shareholder in deterioration of oak growth: common planting practices affect the long-term response of oaks to periodic drought. *For. Ecol. Manage.* 318 133–141. 10.1016/j.foreco.2014.01.017

[B128] Zadworny M. Mucha J. Jagodziński A. M. Kościelniak P. Łakomy P. Modrzejewski M. (2021). Seedling regeneration techniques affect root systems and the response of Quercus robur seedlings to water shortages. *For. Ecol. Manage.* 479:11. 10.1016/j.foreco.2020.118552

[B129] Zhang B. (2015). MicroRNA: a new target for improving plant tolerance to abiotic stress. *J. Exp. Bot.* 66 1749–1761. 10.1093/jxb/erv013 25697792 PMC4669559

[B130] Zhang S. F. Yan S. S. Zhao J. L. Xiong H. H. An P. Q. Wang J. H. (2019). Identification of miRNAs and their target genes in *Larix olgensis* and verified of differential expression miRNAs. *BMC Plant Biol.* 19:20. 10.1186/s12870-019-1853-4 31185902 PMC6558743

[B131] Zhang W. J. Swarup R. Bennett M. Schaller G. E. Kieber J. J. (2013). Cytokinin induces cell division in the quiescent center of the *Arabidopsis* root apical meristem. *Curr. Biol.* 23 1979–1989. 10.1016/j.cub.2013.08.008 24120642

[B132] Zhang W. X. Fan J. J. Tan Q. Q. Zhao M. M. Zhou T. Cao F. L. (2017). The effects of exogenous hormones on rooting process and the activities of key enzymes of *Malus hupehensis* stem cuttings. *PLoS One* 12:13. 10.1371/journal.pone.0172320 28231330 PMC5322878

[B133] Zobel R. W. (2011). A developmental genetic basis for defining root classes. *Crop Sci.* 51 1410–1413. 10.2135/cropsci2010.11.0652

[B134] Zobel R. W. Waisel Y. (2010). A plant root system architectural taxonomy: a framework for root nomenclature. *Plant Biosyst.* 144 507–512. 10.1080/11263501003764483

